# Non-communicable diseases prevention in remote areas of Vietnam: Limited roles of health education and community workers

**DOI:** 10.1371/journal.pone.0273047

**Published:** 2022-09-26

**Authors:** Hang Thi Le, Tuan Anh Le, Tuan Dang Mac, Dua Nhu Nguyen, Ha Ngoc Vu, Anh Thi Mai Truong, Anh Tran Quang Do, Hoai Thi Thu Bui, Huong Thi Thu Do, Anh Thi Hoang Nguyen, Trung Thanh Nguyen, Ngoc The Ngo, Tam Thi Ngo

**Affiliations:** 1 VNU University of Medicine and Pharmacy, Vietnam National University, Hanoi, Vietnam; 2 Hospital of Vietnam National University, Hanoi, Vietnam; 3 Faculty of Medicine, Dai Nam University, Hanoi, Vietnam; Chiang Mai University, THAILAND

## Abstract

**Objective:**

This study aimed to measure the exposure of residents to health education messages about non-communicable diseases (NCD)-related risk factors, and activities of village health workers (VHWs) in NCDs prevention and control in the mountainous setting of Vietnam.

**Method:**

A cross-sectional study was performed in Dap Thanh commune (Ba Che, Quang Ninh province, Vietnam), a mountainous area. There were 151 residents aged 18 years or above recruited for this study. Information regarding exposure to messages about risk factors of NCDs, and activities of VHWs was collected via face-to-face interviews using a structured questionnaire. Multivariate logistic regression was employed to identify associated factors with exposing messages about NCD-related risk factors.

**Results:**

The majority of participants heard about messages related to risk factors of NCDs in the last 30 days, from 56.3% (physical inactivity message), 59.6% (diet message), 75.5% (alcohol use message) to 79.5% (smoking message). Radio/television was the most common source of the messages (from 91.8% to 95.8%) and the majority of participants heard these messages from one source (from 77.1% to 80.9%). Most of sample reported the unavailability of VHWs in their locals (53.6%). Among locals having VHWs, health communication and education was the most common service provided (54.3%); however, only 30% received NCD management services. Participants who had other jobs were less likely to hear about diet-related messages (OR = 0.32; 95%CI = 0.11–0.92), and those ever smoking were more likely to hear these messages in the last 30 days (OR = 6.86; 95%CI = 1.06–44.51). People who had diabetes mellitus were more likely to hear physical activity-related messages in the last 30 days (OR = 2.55; 95%CI = 1.20–5.41).

**Conclusion:**

Our findings indicated that health communication regarding risk factors of NCDs in mountainous areas in Vietnam was insufficient, and the role of health workers as formal information source was not recognized. Efforts should be made to increase the capacity and involvement of VHWs in health education and NCD prevention in mountainous regions.

## Introduction

Non-communicable diseases (NCDs) are a global public health problem that contributes largely to the global burden of diseases [1]. A recent global report shows that NCDs are responsible for more than 41 million deaths each year [2], and this situation is more severe in low- and low-middle-income countries than high-income countries [3]. Common NCDs include cardiovascular disease, cancer, chronic respiratory disease, and diabetes mellius [4]. These diseases are behavioral factors such as tobacco use, drinking alcohol, eating salty, sugar, and fat foods and sedentary lifestyles [2, 4, 5].

Prevention and control of NCDs are possible if people have sufficient knowledge and awareness of the risk factors [2, 4]. Health education, including the health messages communication on risk factors for NCDs, is a common and cost-effective approach to increase public awareness of these factors, which are important to change unhealthy behaviors [6]. Previous reviews showed that NCDs preventive interventions required multisectoral participation, at many levels from the individual, the family, the community and the policy; however, health communication is still considered one of the main components of these interventions [7].

Along with effective health communication campaigns, the involvement of community health workers in NCDs management programs should also be underlined, especially in resource-constrained settings. Community health workers or village health workers (VHWs) are important for countries with limited human resources for health. By mobilizing and task-shifting voluntary residents to become health workers, and offering short-term training to improve their medical knowledge [8, 9], VHWs are expected to fulfill the human resource gaps in addressing the NCDs epidemics [10]. Several reports suggested that VHWs were effective in measuring blood pressure, providing health counseling, managing hypertension, diabetes and other NCDs, as well as offering health education to reduce health risk behaviors [11–14].

Vietnam is one of the countries with an increasing burden of NCDs given its rapid economic growth and urbanization [15]. Previous reports showed that NCDs were responsible for 77% of deaths in Vietnam, in which the most common diseases included cardiovascular diseases, cancer, and diabetes [15]. Vietnam has developed and implemented national strategic programs for NCD prevention since 2002 [16], and currently, the national strategy for the prevention of NCDs in the period 2015–2025 has been issued. This strategy again emphasized the roles of health communication and VHWs [17].

In Vietnam, people living in mountainous areas are vulnerable groups to NCDs because of low education level, low socioeconomic status, limited access to health care due to geographic barriers, and limited availability of service [18, 19]. However, limited evidence about the availability of NCD-related information and roles of VHWs in this setting. This study aimed to measure the exposure of residents to messages about NCD-related risk factors as well as the activities of VWHs in NCDs prevention and control in the mountainous setting of Vietnam. Understanding the availability of health communication on NCD-related risk factors and VHWs in this setting is therefore important to acknowledge gaps that should be fulfilled in future NCD prevention and control plans.

## Methods

### Study design and setting

In July 2019, this survey was conducted in the Dap Thanh commune (Ba Che, Quang Ninh province, Vietnam). This is a typical mountainous area with an area of 91.77 km^2^, a population size of approximately 2000 people, and a population density of approximately 20 people per km^2^. Cross-sectional data were collected from people in the commune. Participants were recruited if they lived in the commune for at least 12 months, aged 18 years or above and were able to answer the face-to-face interview without any support. The formula for estimating a population proportion with specified absolute precision was used to calculate the required sample size, with expected proportion = 0.5 (according to the previous study in Vietnam [20]), confidence level = 95%, absolute precision = 0.08, resulting in a sample size of 151 residents. A sample frame was developed with the support of the local authorities, and the local health staff and the research team identified who were eligible for the study. After that, a random sampling technique was used to randomly select the participants in the sampling frame. Then, invitations were sent to them, and if they refused to participate in the study, people who were next to them in the list were invited. The final sample size for analysis was 151 people. The study protocol was approved by the Institutional Review Board of the School of Medicine and Pharmacy, Vietnam National University (Code: QD 05/2018).

### Variables and measurement

Participants were invited to go to the local commune health station for physical examination and a face-to-face interview. They were first examined by the physician in the research team to identify which health problems they currently suffered at the time of the data collection, and then, were interviewed by the data collectors who were undergraduate medical students of the University of Medicine and Pharmacy, Vietnam National University. Both physicians and medical students were undergone training sessions to understand the examination data collection processes and ensure that these processes were conducted consistently across participants.

A structured questionnaire was used for the interview. Questions regarding demographic characteristics (age, gender, ethnicity, education level, occupation, and monthly household income) and behaviors (frequency of alcohol use and smoking habits) were asked. Data about health status, activities of VHWs and exposure to messages about risk factors of NCDs were also collected.

#### Health indicators

Systolic and diastolic blood pressures were evaluated twice, and the final result was the mean of the two evaluations. People were classified to have hypertension if they had a systolic blood pressure level of ≥140 mmHg and/or diastolic blood pressure level ≥ of 90 mmHg [21]. Fasting blood glucose level was assessed by taking blood samples and then sending them to the Vietnam National University hospital for measurement after the field trip. Testing results were then sent back to the residents after completely being analyzed. Residents with a blood glucose level of ≥ 7.5 mmol/L [22] were determined as diabetes mellitus patients. All hypertensive and diabetic patients were recommended to seek health care for disease management. Participants were also measured height and weight. Body mass index was then calculated and was used to define normal (18.5–22.9 kg/m^2^); underweight (< 18.5 kg/m^2^) and overweight/obesity (≥ 23 kg/m^2^) conditions based on the Asian populations’ references [23].

#### Exposure to messages about risk factors of NCDs

Participants were asked whether they heard the following messages in the past 30 days, the number of times that they heard these messages, and from which information sources (Health workers/Family members/Friends/relatives/ Radio/Television/ Internet):

- Smoking increases the risk of getting NCDs
- Abusing alcohol increases the risk of getting NCDs.
- A high fat/salt/sugar diet increases the risk of getting NCDs
- Physical inactivity increases the risk of getting NCDs.

#### Information about activities of VHWs

Information was collected about the availability of VHWs in the local, frequency of VHWs’ visits to household, which services they provided according to the following services (based on the requirement of the Ministry of Health):

- Health communication and education in the community
- Guidance on hygiene to prevent diseases
- Maternal and child health care and family planning
- First aid and routine care
- Participate in implementing health programs in villages
- Managing chronic diseases such as hypertension, diabetes mellitus

### Statistical analysis

Mean, standard deviation, frequency, and percentage were used for descriptive statistics. Multivariate logistic regression was used to examine factors related to the exposure to different NCD-related messages in the last 30 days. Independent variables included demographic characteristics, health indicators and availability of VHWs in the local. A p-value < 0.05 was utilized for defining statistical significance. Stata software 15.0 was used for analysis.

## Results

Of 151 participants, Table 1 summarizes sociodemographic characteristics of the participants. Most of them were female, aged 60 years or more, belonged to Tay and san Chi ethnics, having primary education or below and worked as farmers/blue-collar workers. The mean monthly household income was 1.4 million VND (SD = 1.5). The majority of the sample did not use alcohol or smoke. The rates of diabetes mellitus, hypertension and overweight/obesity were 53.0%, 32.5% and 37.1%, respectively.

**Table 1:** Demographic characteristics of participants (n = 151).

Characteristics	Values
Gender, Female, n(%)	104 (68.9%)
Age, ≥ 60 years old, n(%)	46 (30.5%)
Ethnicity, n(%)	
Tay	47 (31.1%)
San Chi	55 (36.4%)
Dao	28 (18.5%)
Can Lan	15 (9.9%)
Others	6 (4.0%)
Education level, n(%)	
Illiteracy/Primary school	117 (77.5%)
Secondary school	28 (18.5%)
High school or above	6 (4.0%)
Occupation, n(%)	
Farmers/Blue-collar workers	126 (83.4%)
Others	25 (16.6%)
Monthly household income (million VND), Mean (SD)	1.4 (1.5)
Frequency of alcohol use, n(%)	
Never	113 (74.8%)
Monthly or less	21 (13.9%)
2–4 times per month	9 (6.0%)
2–3 times per week	3 (2.0%)
≥ 4 times per week	5 (3.3%)
Smoking habit, n(%)	
Never	136 (90.1%)
Former smoker	6 (4.0%)
Irregular smoker	3 (2.0%)
Regular smoker	6 (4.0%)
Having diabetes mellitus, n(%)	80 (53.0%)
Having high blood pressure, n(%)	49 (32.5%)
Body mass index categories, n(%)	
Normal (18.5–22.9 kg/m^2^)	85 (56.3%)
Underweight (< 18.5 kg/m^2^)	10 (6.6%)
Overweight/obesity (≥ 23 kg/m^2^)	56 (37.1%)

Table 2 depicts that the majority of the participants heard about messages related to risk factors of NCDs in the last 30 days, from 56.3% (for physical inactivity message) to 79.5% (for smoking message). Radio/television was the most common source of the messages (from 91.8% to 95.8%) and the majority of participants hearing these messages from one source (from 77.1% to 80.9%).

**Table 2:** Exposure to messages about NCD-related risk factors among participants (n = 151).

Characteristics	Messages			
	Smoking increases the risk of getting NCDs.	Abusing alcohol increases the risk of getting NCDs.	A high fat/salt/sugar diet increases the risk of getting NCDs	Physical inactivity increases the risk of getting NCDs.
Heard in the last 30 days, n(%)	120 (79.5%)	114 (75.5%)	90 (59.6%)	85 (56.3%)
Number of exposure times in the last 30 days, Mean (SD)	3.4 (3.8)	3.4 (3.5)	2.6 (2.9)	2.2 (2.5)
Sources of information, n(%)				
Health workers	19 (15.8%)	17 (14.9%)	13 (14.4%)	13 (15.3%)
Family members	6 (5.0%)	6 (5.3%)	1 (1.1%)	3 (3.5%)
Friends/relatives	5 (4.2%)	3 (2.6%)	3 (3.3%)	5 (5.9%)
Radio/Television	115 (95.8%)	110 (96.5%)	94 (93.3%)	78 (91.8%)
Internet	9 (7.5%)	8 (7.0%)	7 (7.8%)	7 (8.2%)
Number of information sources, n(%)				
One	93 (77.5%)	90 (79.0%)	72 (80.9%)	64 (77.1%)
Two	21 (17.5%)	19 (16.7%)	15 (16.9%)	15 (18.1%)
Three or more	6 (5.0%)	5 (4.3%)	2 (2.2%)	4 (4.8%)

Most of the participants reported the unavailability of VHWs in their local. Among locals having VHWs, Table 3 shows that the most common service provided was health communication and education (54.3%), following by guidance on hygiene to prevent diseases (47.1%) and maternal and child healthcare, and family planning (40.0%).

**Table 3:** Activities of village health workers.

Characteristics	Values
VHWs are available in the local, n(%) (n = 151)	70 (46.4%)
Number of visits of VHWs in the last 12 months, n(%) (n = 70)	
None	25 (35.7%)
Once	18 (25.7%)
Twice	12 (17.1%)
More than twice	8 (11.4%)
Don’t know/Don’t remember	7 (10.0%)
Service utilization (n = 70)	
Health communication and education	38 (54.3%)
Guidance on hygiene to prevent diseases	33 (47.1%)
Maternal and child health care and family planning	28 (40.0%)
First aid and routine care	21 (30.0%)
Participate in implementing health programs in villages	21 (30.0%)
Managing NCDs such as hypertension, diabetes mellitus	21 (30.0%)

* VHWs: Village health workers

Table 4 indicates that none of the factors were associated with hearing about smoking- and alcohol-related messages in the past 30 days. Meanwhile, participants who had other jobs were less likely to hear about diet-related messages (OR = 0.32; 95%CI = 0.11–0.92); whereas, those ever smoking were more likely to hear these messages in the last 30 days (OR = 6.86; 95%CI = 1.06–44.51). People who had diabetes mellitus were more likely to hear physical activity-related messages in the last 30 days (OR = 2.55; 95%CI = 1.20–5.41). Notably, the availability of VHWs was not associated with the exposure to these messages.

**Table 4:** Factors associated with exposing messages on NCDs risk factors.

Characteristics	Heard about smoking-related message	Heard about alcohol-related message	Heard about diet-related message	Heard about physical activity-related message
	OR (95%CI)	OR (95%CI)	OR (95%CI)	OR (95%CI)
Gender				
Male	1	1	1	1
Female	0.81 (0.23–2.92)	0.81 (0.24–2.69)	0.59 (0.19–1.78)	0.96 (0.31–3.03)
Age (per year)	1.00 (0.97–1.03)	1.00 (0.97–1.03)	1.01 (0.98–1.04)	0.99 (0.97–1.02)
Education				
Illiterate/Elementary	1	1	1	1
Secondary or above	2.27 (0.67–7.72)	1.64 (0.55–4.84)	2.56* (0.95–6.87)	2.62* (0.97–7.06)
Occupation				
Farmers/Blue-collar workers	1	1	1	1
Others	0.75 (0.21–2.69)	0.59 (0.19–1.88)	0.32** (0.11–0.92)	0.53 (0.18–1.53)
Alcohol use				
No	1	1	1	1
Yes	1.07 (0.27–4.25)	1.37 (0.36–5.22)	1.21 (0.37–3.99)	1.12 (0.33–3.81)
Ever smoking				
No	1	1	1	1
Yes	-	-	6.86** (1.06–44.51)	6.00* (0.92–38.94)
Having diabetes				
No	1	1	1	1
Yes	1.33 (0.55–3.24)	1.41 (0.61–3.25)	1.70 (0.81–3.58)	2.55** (1.20–5.41)
Having hypertension				
No	1	1	1	1
Yes	1.21 (0.39–3.76)	0.95 (0.34–2.64)	1.67 (0.65–4.32)	1.18 (0.47–2.98)
Body mass index (per kg/m^2^)	1.05 (0.88–1.24)	1.03 (0.89–1.21)	1.01 (0.89–1.15)	0.89* (0.77–1.02)
Available village health workers in local				
No	1	1	1	1
Yes	1.44 (0.59–3.53)	1.33 (0.57–3.10)	0.81 (0.38–1.72)	1.07 (0.50–2.29)

*p<0.1;

**p<0.05;

***p<0.01

## Discussion

Our research provides preliminary evidence on the gaps related to NCDs prevention in the mountainous regions of Vietnam. Results showed that health communication and education for NCD prevention in mountainous areas of Vietnam was still limited. Although messages about the harmful effects of alcohol and tobacco were common among study participants, only more than half of the population had ever heard messages about diet and physical activity in the last 30 days before the interview. In addition, the study indicated the shortage of VWHs, as well as VWHs had not played a significant role in the prevention and management of NCDs in the mountainous areas.

Results of the current study indicated that messages related to alcohol use and smoking in mountainous areas were prevalent. This could be explained that in recent times, national tobacco and alcohol use prevention programs have been strongly deployed nationwide. This promoted the dissemination of information through mass media such as television or radio; hence, more people knew about this information. Multivariate model results also found no relationship between the reception of this information with other factors. This demonstrates that this information has been widely disseminated and did not differentiate among people in mountainous regions regardless of socioeconomic characteristics and medical conditions. However, information related to diet and physical activity is not well disseminated since study results showed that only more than half of the study sample heard this information within the past 30 days, which was consistent with the previous study in Vietnam [20]. In addition, the regression model showed that people with risk factors such as smoking, or those who already have NCDs such as diabetic mellitus patients were more likely to be exposed to this information. As such, information about diet and physical activity was not widely available, and only people with specific risk factors or health conditions such as diabetes were beneficial from these media campaigns.

Television/radio was the main source of information with over 90% of the people using these tools to listen to health messages. This result is similar to studies in Vietnam [20] and Japan [24], but different from results in Hong Kong where people preferred newspapers/magazines [25], or results in Nigeria when medical staff was found as the main source of health information [26]. On the other hand, our results also showed that the role of health workers in providing health messages was not significant when only approximately 15% of people received information from this source. Thus, although the grassroots health system network in Vietnam has been well organized with 99% of communes having health stations and 79% of villages having VHWs [27], and health workers were the formal information source for disseminating health knowledge, health communication about risk factors from health workers in mountainous areas seems to be insufficient in terms of both quantity and content of messages.

Previous reports suggested that community health workers or VHWs could play an important role in preventing and managing NCDs in the community via approaching high-risk people, providing counseling, referring services, monitoring care and others [28–30]. In Nepal, a mountainous country, female community health workers have been proven as an important connection between health care facilities and communities [31]. Moreover, if they received appropriate training, they could deliver hypertension management program along with maternal care [31]. The Vietnam national strategy emphasized the role of VHWs in NCD prevention and control [17]. However, our research showed that VHWs played a minor role in the prevention and management of NCDs. First, regarding the quantity aspect, although our sample includes people in all the villages of Dap Thanh commune, only half of our sample reported having VHWs in their community. Second, in villages with VHWs, activities related to NCDs such as health education and communication, and the management of NCDs such as diabetes or hypertension were also limited. This result suggests that there is a need for more training programs to help build the capacity of VHWs, as well as mechanisms to help recruit more VHWs in communities that do not yet have VHWs. Thus, the national system of prevention and care of NCDs ensures access to the entire population, especially people living in mountainous areas. Unfortunately, our study did not have data about facilitators and barriers toward activities of VHWs in NCDs management. Further studies should be warranted to examine the role of VHWs in NCDs prevention and care in the view of VHWs.

Several limitations should be noted. First, the cross-sectional design was used in this study. This reduces the possibility of establishing causal relationships among variables. Second, the study was conducted in a mountainous commune with small sample size; hence, the ability to extrapolate populations of other mountainous regions is limited. Third, the study uses a structured questionnaire to interview information during the 30 days prior to the interview, which could lead to recall bias.

## Conclusions

Findings indicated that health communication regarding risk factors of NCDs in mountainous areas in Vietnam was insufficient, and the role of health workers as a formal information source was not recognized. VHWs were inadequately involved in NCDs prevention and management. Efforts should be made to increase the capacity and involvement of commune health workers in health education and NCD prevention in mountainous regions.

## Supporting information

S1 File Dataset of this study. (DTA)
